# Upper-limb magnetic resonance lymphangiography: a useful new technique

**DOI:** 10.1590/0100-3984.2018.0067

**Published:** 2019

**Authors:** Luís Felipe Fiorentini, Guilherme Stüker, Gabriel Sartori Pacini, Edson Marchiori, Bruno Hochhegger

**Affiliations:** 1 Department of Diagnostic Methods, Pontíficia Universidade Católica do Rio Grande do Sul (PUCRS), Porto Alegre, RS, Brazil.; 2 Medical Imaging Research Lab (Labimed), Hospital Pavilhão Pereira Filho, Santa Casa de Misericórdia de Porto Alegre, Porto Alegre, RS, Brazil.; 3 Universidade Federal do Rio de Janeiro (UFRJ), Rio de Janeiro, RJ, Brazil.; 4 Universidade Federal de Ciências da Saúde de Porto Alegre (UFCSPA), Porto Alegre, RS, Brazil.

## INTRODUCTION

Recent studies have highlighted the importance of magnetic resonance imaging in the evaluation of diseases affecting the musculoskeletal system^(1-6)^ . Contrast-enhanced magnetic resonance lymphangiography (MRL) is a recently developed minimally invasive imaging technique for the evaluation of the lymphatic system and its abnormalities. Lymphedema is one of the most widely studied diseases affecting the lymphatic vessels of the upper and lower limbs^(7,8)^; it can be classified as primary (resulting from congenital malformations in vascular morphogenesis, which can be symptomatic later in life) or secondary (mainly resulting from trauma, infection, or malignant tumors).

Novel microsurgical procedures, such as the use of lymphovenous shunts, have allowed major advances to be made in the treatment of lymphedema and lymphatic diseases in general. Simultaneously, the development of new imaging techniques has allowed physicians to identify the anatomical and pathological features of the lymphatic system in order to define the treatment and plan the surgical procedure. In that context, MRL represents an appealing option, because it produces images with high spatial and temporal resolution through the use of three-dimensional enhancement techniques, as well as because, unlike other imaging methods, it does not expose the patient to ionizing radiation^(7,8)^ .

Although MRL shows great promise, there are as yet few data available regarding its technical application. In addition, most of the MRL studies conducted to date have focused on the lower limbs, addressing the upper limbs only in a secondary evaluation. Therefore, the objective of the present study was to examine the techniques employed in the upper-limb MRL procedure, demonstrating the main points of the examination and its particularities.

## UPPER-LIMB MRL PROCEDURE

**Figure 1:**
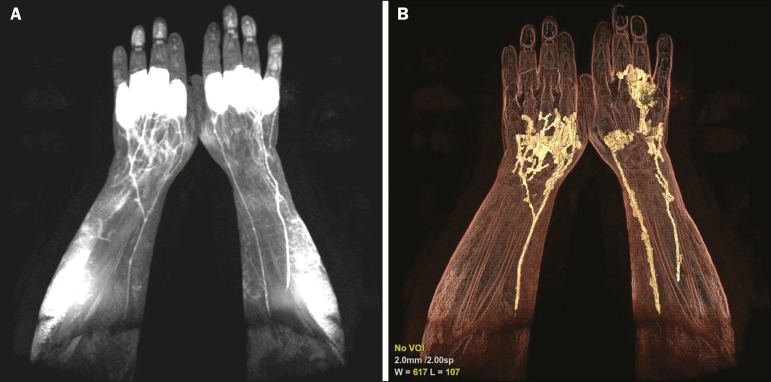
A 54-year-old man with rheumatoid arthritis. **A:** Maximum intensity projection of a volumetric T1-weighted sequence, with a slice thickness of 1 mm, showing normal lymphatic vessels in both upper limbs, without signs of obstruction. **B:** The same acquisition with three-dimensional enhancement techniques.

Initially, the patient should be evaluated to determine if there is any contraindication to performing magnetic resonance imaging. Eligible patients should be provided with a full, detailed explanation about the procedure, including its benefits and risks, and written informed consent should be obtained from each patient. After local asepsis, 1-2 mL of a mixture of contrast media and local anesthetic are injected subcutaneously into each interdigital space in both hands simultaneously. Typically, a mixture of 0.5 mL 2% lignocaine and 4.5 mL of gadobutrol (Gadovist; Bayer Schering Pharma, Berlin, Germany) is administered at a dose of 0.1 mmol/kg body weight. The injection site is then massaged for 2 min, after which the scan is started. The anesthetic allows the patient to tolerate the procedure, and the contrast media will be continuously drained by the lymphatic system, providing the required information about its integrity/abnormalities. The patient is placed in the prone position, the upper limbs entering the scanner first, allowing the evaluation of the hands, wrists, forearms, elbows, upper arms, and shoulders, in that order. The use of cushions between the coil and the skin can reduce artifacts. Although it is possible to use a 1.5 T scanner, better results are obtained with a scanner that has a magnetic field strength of 3.0 T or higher^(7,8)^. The MRL protocol included the following: an electrocardiogram-gated steady-state free precession sequence-slice thickness 5 mm, field of view (FOV) 40 × 40 mm, matrix 256 × 256, bandwidth 125 kHz/pixel, and repetition time/echo time (TR/TE) 3.1/1.6 ms; a T1-weighted sequence with spectral inversion at lipids-flip angle 25º, FOV 44 × 44 mm, matrix 256 × 180, slice thickness 3 mm, bandwidth 110 MHz, and TR/TE 3.7/2.1 ms; and a 3D T2-weighted turbo spin-echo sequence-FOV 40 × 40 mm, matrix 320 × 224, slice thickness 4 mm, bandwidth 32 MHz, and TR/TE 4670/113 ms. As an example, we present the MRL imaging obtained in the case of a 54-year-old male patient, previously diagnosed with rheumatoid arthritis, who presented with a 5-year history of lymphedema in the upper limbs (Figure 1).

## CONCLUSION

MRL is a safe, noninvasive imaging method that provides detailed anatomical and functional information regarding the lymphatic system, which is particularly useful in patients with lymphedema. Therefore, it represents a promising tool for the diagnosis and treatment of lymphatic disorders.
